# Supplementary material for: Park7 deletion leads to sex-specific transcriptome changes involving NRF2-CYP1B1 axis in mouse midbrain astrocytes
Source: NPJ Parkinsons Dis. 2025 Jan 4;11:8. doi: 10.1038/s41531-024-00851-7 (PMC11700157; doi:10.1038/s41531-024-00851-7)

# *Park7* deletion leads to sex-specific transcriptome changes involving NRF2-CYP1B1 axis in mouse midbrain astrocytes

## Supplementary Information

**Supplementary Figures**

**Supplementary Table Legends**

**Supplementary Information**

The Supplementary Information contains seven Supplementary Figures and five Supplementary Tables.

**Supplementary Figure 1. Distribution of the per-gene dispersion in *Park7*$^{-/-}$ mouse midbrain.** Per-gene dispersions to estimate the median dispersion each sample set show similar results in all sample sets, except for the second cohort of 8-month-old male samples. Lack of changes in 3 and 8-month-old females are demonstrated to not be due to technical or biological (oestrus cycle) variation between the female samples. Increased dispersion in the second cohort of 8-month-old male samples partially explains the lower number of DEGs compared to the first cohort.

**Supplementary Figure 2. Loss of *Park7* in female mice does not lead to gene expression changes associated with NRF2 signaling, epithelial-to-mesenchymal transition, focal adhesion, and extracellular matrix composition.** (a-d) Pathway enrichment analysis for the 101 DEGs altered in 8-month-old female mice. The top 5 results from (a) KEGG pathways, (b) WikiPathway pathways, (c) MSigDB Hallmark pathways, and (d) GO terms for cellular components based on the significance of enrichment are shown.The x-axis represents the -log10 p-value of pathway enrichment. Complete results of enrichment analysis are available in the Supplementary Table S4. (e) Top 5 TFs associated with DEGs from Park7-/- female mice based on the significance of enrichment for primary TF targets from the ENCODE project and ChEA database. The x-axis represents the -log10 p-value. (f) Top5 Upstream Regulators associated with DEGs from Park7-/- female mice predicted by Ingenuity Pathway Analysis (IPA). The x-axis represents the -log10 p-value of pathway enrichment.

**Supplementary Figure 3. RNAscope and RT-qPCR validation of gene expression levels. (a-b)** RNAscope validation of *Cdh1* expression in the (a) midbrain of *Park7*$^{-/-}$ female mice and (b) cortex of *Park7*$^{-/-}$ male mice at 8-months of age. *Cdh1* mRNA is stained in purple, DAPI staining of DNA is in blue. Representative images from analysis of 3 independent mice per genotype are shown. No differences were observed. **(c-d)** RT-qPCR analysis of (c) *Irs2* and (d) *Crim1* expression in the midbrain of *Park7*$^{-/-}$ male and female mice and in male cortex at 8-months of age. Values represent mean ± SEM (n=4-8 mice per group). Statistical significance was tested by unpaired t-test. * = p-value < 0.05. **(e-f)** RNAscope validation of *Gstm2* expression in the (a) midbrain of *Park7*$^{-/-}$ female mice and (b) cortex of *Park7*$^{-/-}$ male mice at 8-months of age. *Gstm2* mRNA is stained in purple, DAPI staining of DNA is in blue. Representative images from analysis of 3 independent mice per genotype are shown. No differences were observed.

**Supplementary Figure 4. Analysis of Cyp1b1 expression.** (**a-b)** RNAscope validation of *Cyp1b1* expression in the (a) midbrain of *Park7*$^{-/-}$ female mice and (b) cortex of *Park7*$^{-/-}$ male mice at 8-months of age. *Cyp1b1* mRNA is stained in red, DAPI staining of DNA is in blue. Representative images from analysis of 3 independent mice per genotype are shown. No differences were observed. **(c)** Immunohistochemistry of CYP1B1 in mouse kidney sections was used to test the specificity of the CYP1B1 antibody. CYP1B1 is stained in red, DRAQ7 staining of DNA is in purple.

**Supplementary Figure 5. Genotyping of newborn mice prior to glial cell isolation.** *Ube* primers were used to test whether DNA from newborn mouse tails carried XY (two separate bands) or XX (one stronger band) genotype. L = Ladder.

**Supplementary Figure 6. Expression analysis of cell type-purity and astrocyte reactivity marker genes in primary glial cultures. (a-b)** Expression levels of the indicated **(a)** cell type-specific marker genes in cultures of isolated primary astrocytes, microglia, and oligodendrocytes, and **(b)** markers of reactive astrocytes in primary astrocytes and microglia, as determined by RNA-seq analysis. Red dots represent female and blue dots male samples, asterisk indicates the mean of three independent samples, and x-axis represents transcripts per million (TPM).

**Supplementary Figure 7. Enrichment analysis of upregulated genes upon *Park7* knock-down. (a-c)** Heatmaps showing the differences in pathway enrichments of upregulated DEGs from **panels a-c** using the (**a)** ENCODE project and ChEA databases, **(b)** BioPlanet, and **(c)** MSigDB Hallmark databases, focusing on the top 5 most enriched pathways or TFs upon *Park7* depletion. The -$\log_{10}$ p-values of the enrichments are depicted as color scale.

**Supplementary Table 1. List of DEGs from *Park7*$^{-/-}$ mice compared to wild type littermate controls.** The separate worksheets include DEGs from 3-month-old female mice, 3-month-old male mice, 8-month-old female mice, 8-month-old male mice (first cohort), 8-month-old male mice (second cohort), 8-month-old male mice (first and second cohort combined using batch correction), with FDR < 0.05. The table includes ENSEMBL IDs, base mean, $\log_2$-fold change, p-value, FDR (padj), and gene symbol for each DEG.

**Supplementary Table 2. Mapped reads for the 4 groups of mice and the 3 groups of astrocytes.**

**Supplementary Table 3. Lists of enriched pathways in males.** Separate worksheets include enrichments for analysis using DEGs obtained from the comparison of 8-month-old male *Park7*$^{-/-}$ mice with the wild type littermate controls (first and second cohort together) from KEGG, WikiPathway, MSigDB, and BioPlanet databases, and the GO terms for Cellular components.

**Supplementary Table 4. Lists of enriched pathways in females.** Separate worksheets include enrichments for analysis using DEGs obtained from the comparison of 8-month-old female *Park7*$^{-/-}$ mice with the wild type littermate controls from KEGG, WikiPathway, MSigDB, and BioPlanet databases, and the GO terms for Cellular components.

**Supplementary Table 5. Lists DEGs in primary mouse astrocytes upon the knock-down of *Park7*, *Nfe2l2*, or *Cyp1b1*.** DEGs were defined by applying FDR < 0.05. The table includes ENSEMBL IDs, base mean, $log_2$-fold change, p-value, FDR (padj), and gene symbol for each DEG.

# Supplementary Figure 1

**a**

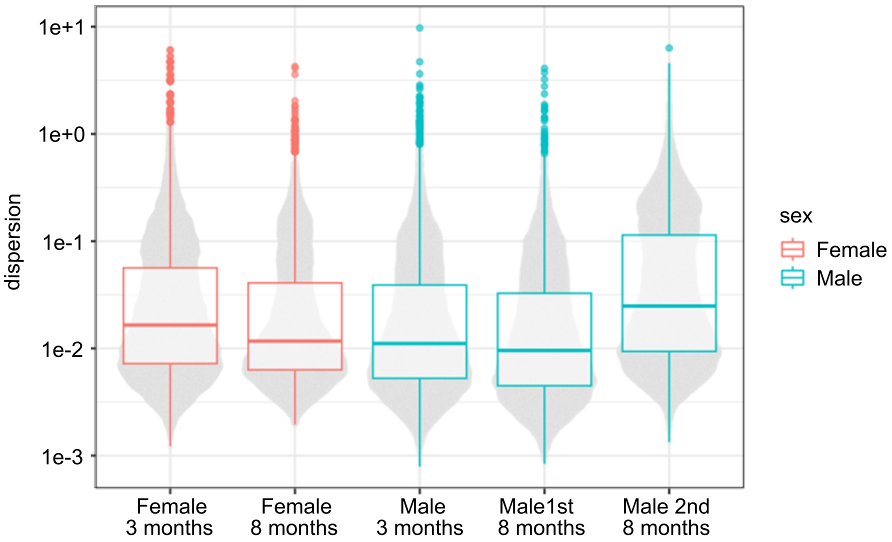

# Supplementary Figure 2

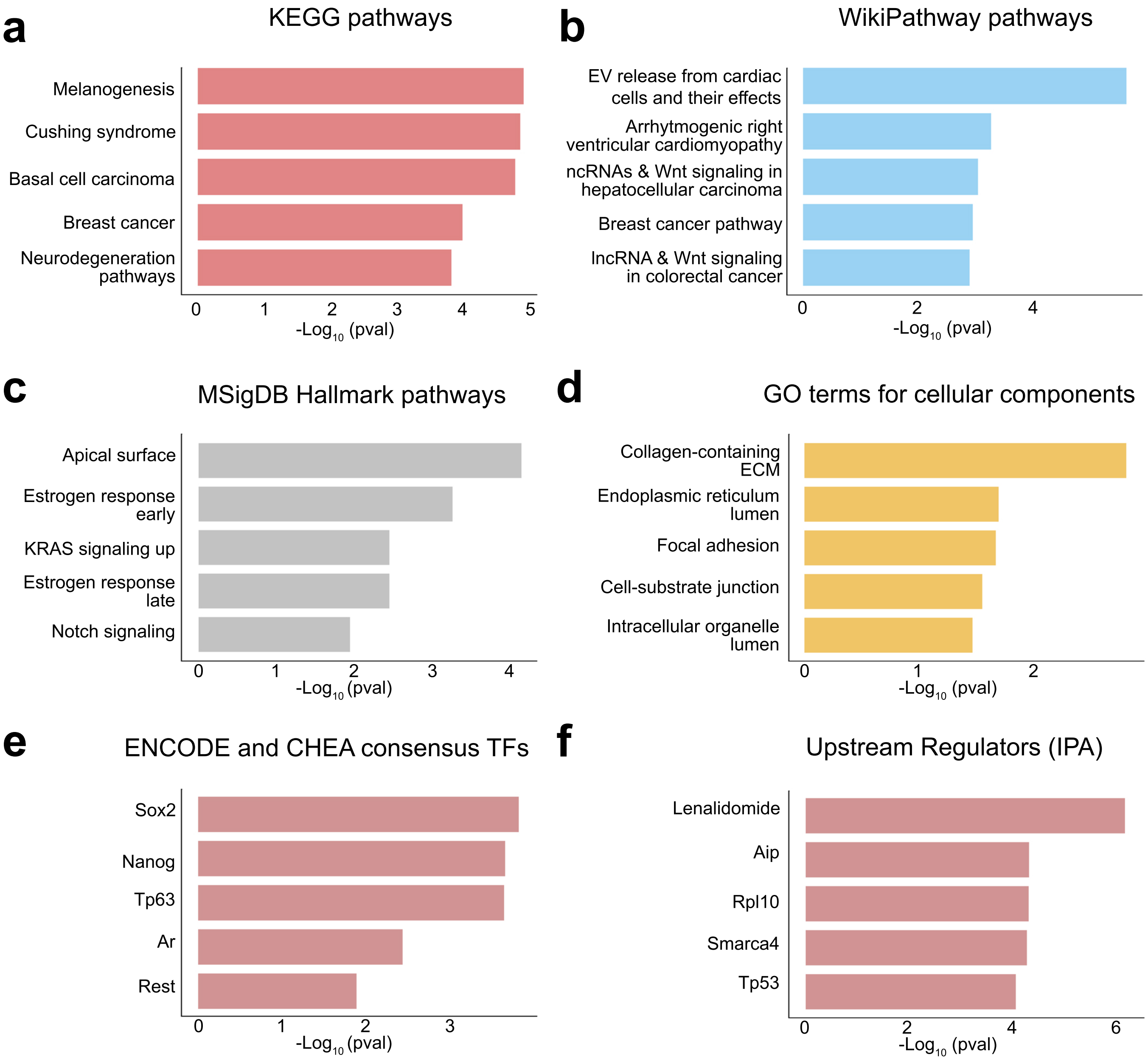

# Supplementary Figure 3

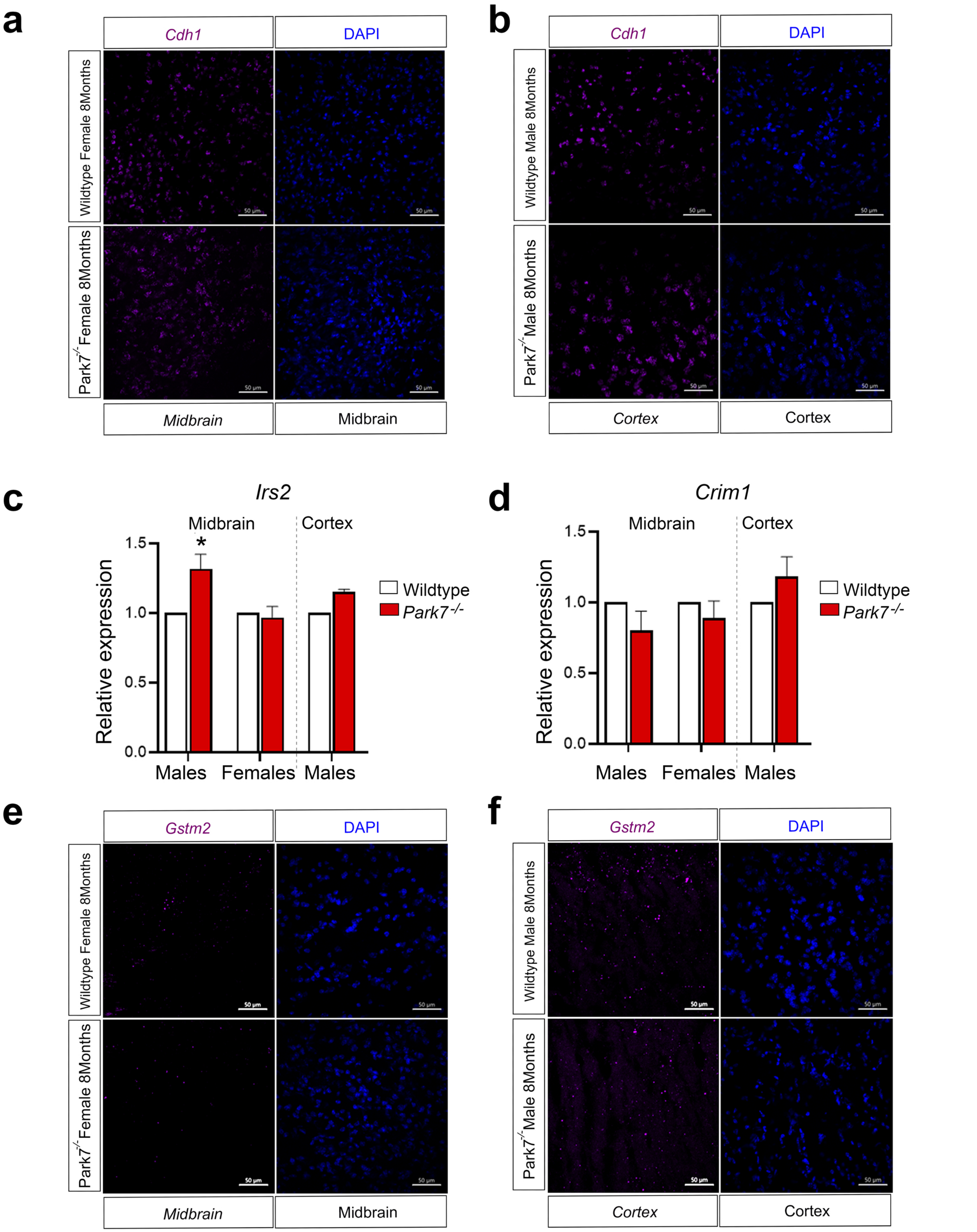

# Supplementary Figure 4

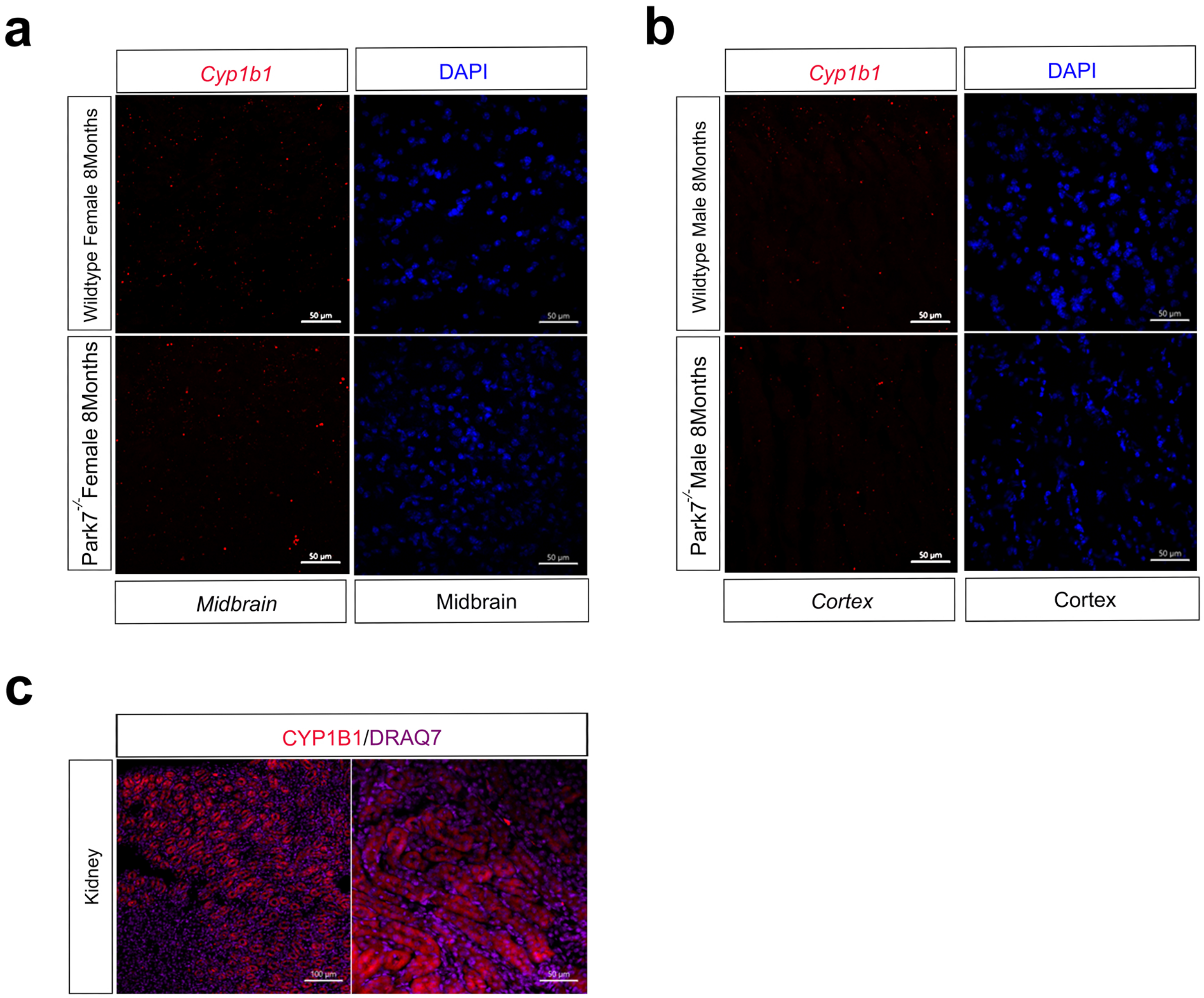

# Supplementary Figure 5

**a**

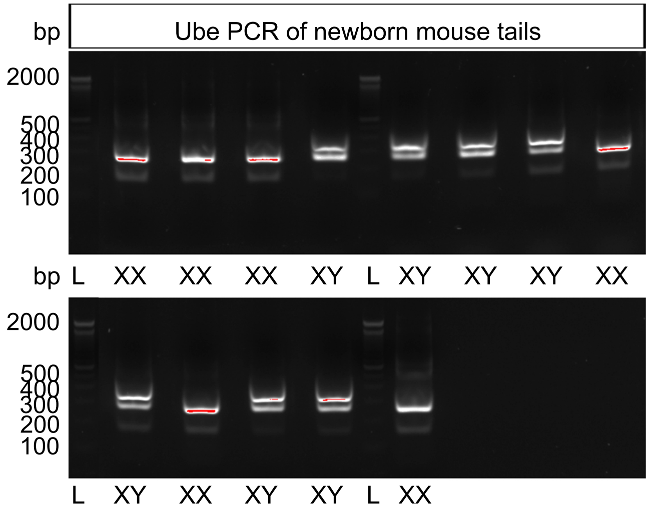

# Supplementary Figure 6

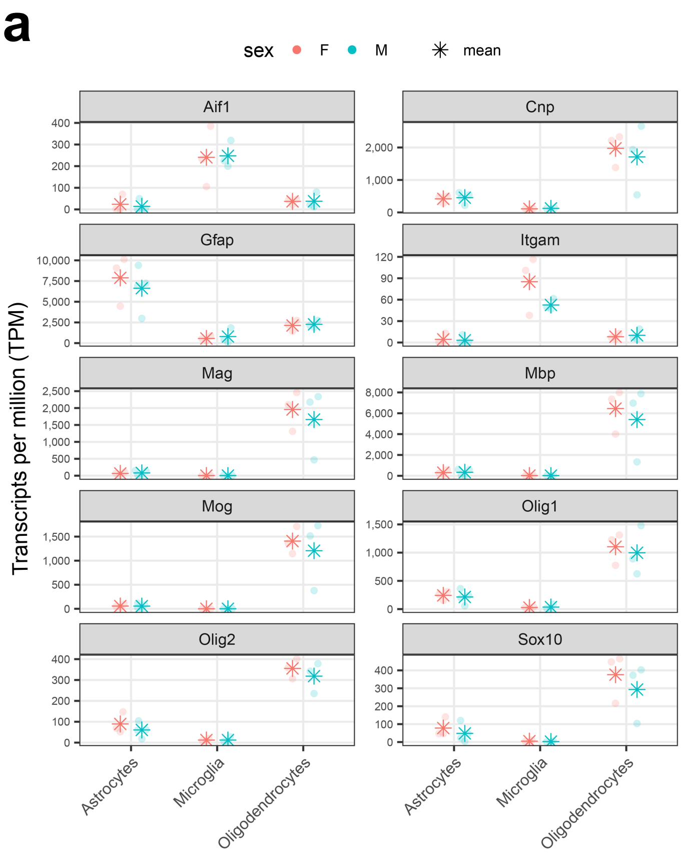


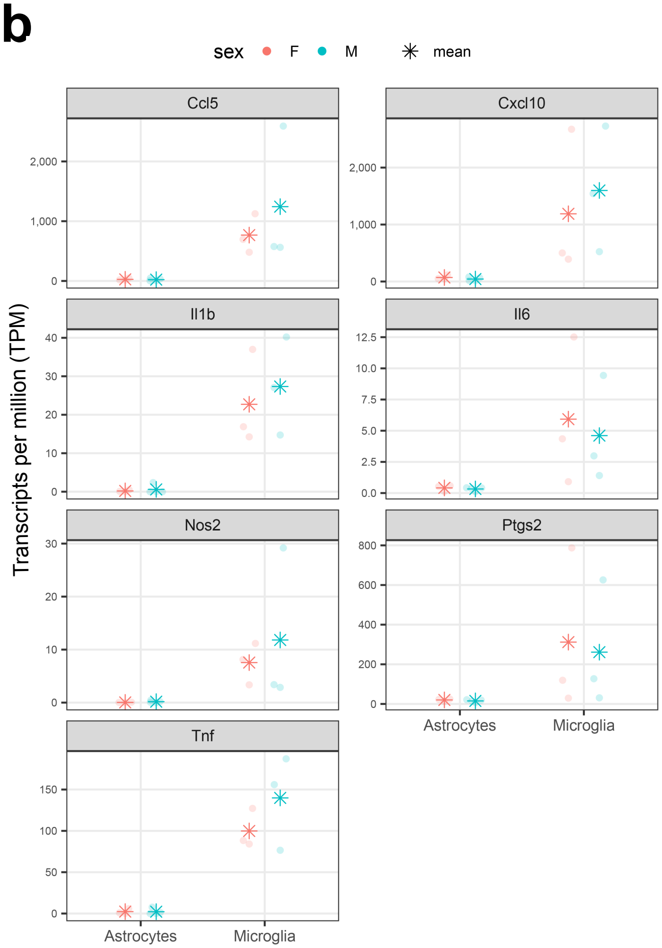

# Supplementary Figure 7

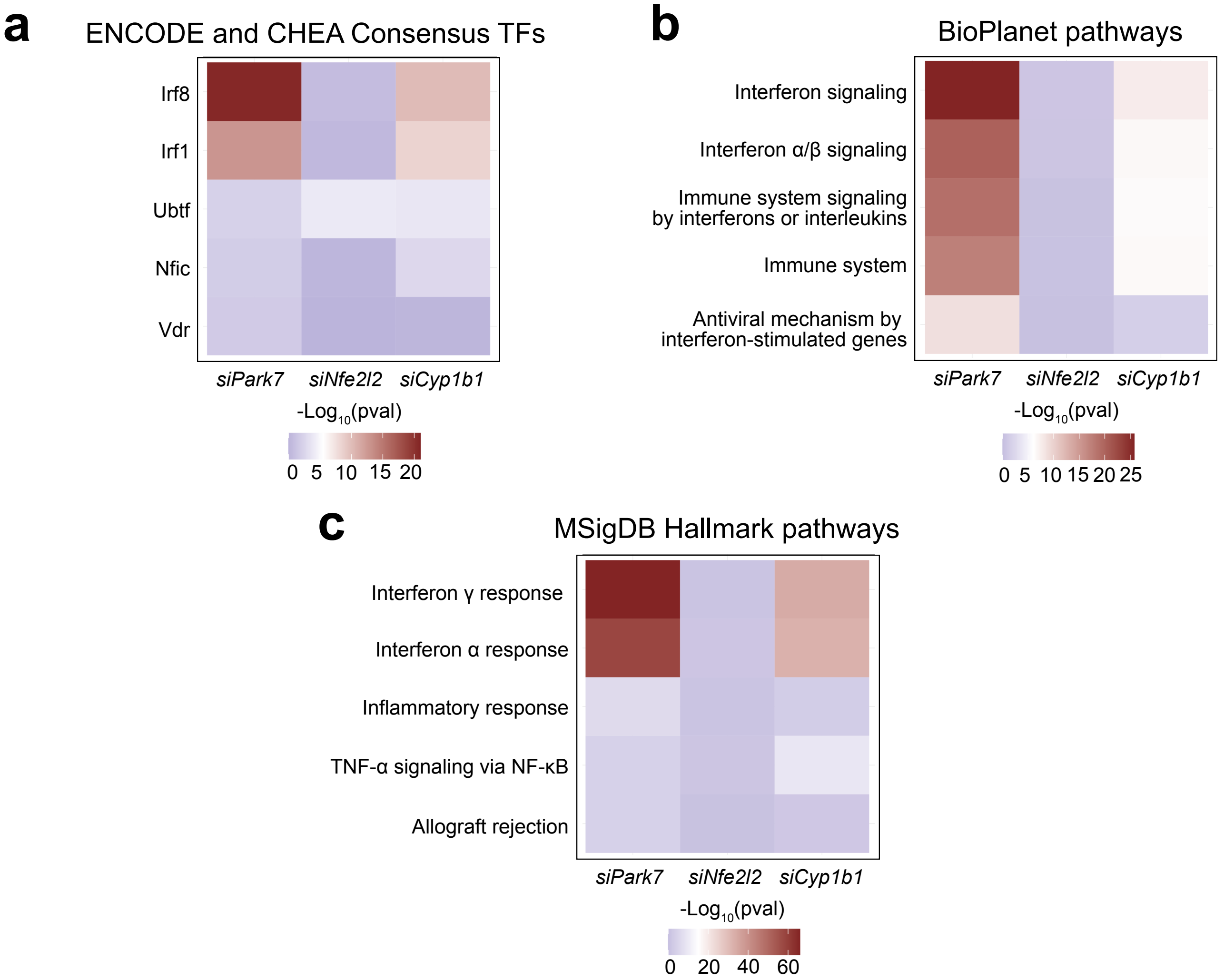

Supplement: Supplementary file 1 — Supplementary Material [file 41531_2024_851_MOESM1_ESM.pdf]
